# COVID-19 Vaccine Acceptance and Hesitancy in Health Care Workers in Somalia: Findings from a Fragile Country with No Previous Experience of Mass Adult Immunization

**DOI:** 10.3390/vaccines11040858

**Published:** 2023-04-17

**Authors:** Abdulrazak Mohamed Ibrahim, Mohammad Hamayoun, Muhammad Farid, Umar Al-Umra, Mukhtar Shube, Kyandindi Sumaili, Lorraine Shamalla, Sk Md Mamunur Rahman Malik

**Affiliations:** 1 World Health Organization, Mogadishu 63565, Somalia; 2 Federal Ministry of Health and Human Services, Mogadishu P.O. Box 22, Somalia; 3 United Nations Children’s Fund, Mogadishu 44145, Somalia

**Keywords:** COVID-19 vaccine, vaccine hesitancy, acceptance, healthcare workers, Somalia

## Abstract

Coverage of COVID-19 vaccines in Somalia remains low, including among health workers. This study aimed to identify factors associated with COVID-19 vaccine hesitancy among health workers. In this cross-sectional, questionnaire-based study, 1476 health workers in government and private health facilities in Somalia’s federal member states were interviewed face-to-face about their perceptions of and attitudes toward COVID-19 vaccines. Both vaccinated and unvaccinated health workers were included. Factors associated with vaccine hesitancy were evaluated in a multivariable logistic regression analysis. Participants were evenly distributed by sex, and their mean age was 34 (standard deviation 11.8) years. The overall prevalence of vaccine hesitancy was 38.2%. Of the 564 unvaccinated participants, 39.0% remained hesitant. The factors associated with vaccine hesitancy were: being a primary health care worker (adjusted odds ratio (aOR) = 2.37, 95% confidence interval (CI): 1.15–4.90) or a nurse (aOR = 2.12, 95% CI: 1.05–4.25); having a master’s degree (aOR = 5.32, 95% CI: 1.28–22.23); living in Hirshabelle State (aOR = 3.23, 95% CI: 1.68–6.20); not having had COVID-19 (aOR = 1.96, 95% CI: 1.15–3.32); and having received no training on COVID-19 (aOR = 1.54, 95% CI: 1.02–2.32). Despite the availability of COVID-19 vaccines in Somalia, a large proportion of unvaccinated health workers remain hesitant about being vaccinated, potentially influencing the public’s willingness to take the vaccine. This study provides vital information to inform future vaccination strategies to achieve optimal coverage.

## 1. Introduction

The COVID-19 pandemic caused severe and widespread disruption to essential health services in Somalia, even two years after the first case of COVID-19 was detected in the country on 16 March 2020 [1]. The reasons for this disruption include the lockdown and closure of health facilities because of absenteeism of health care workers who feared getting COVID-19. As a result, the country’s routine immunization service has been interrupted, resulting in thousands of children missing routine immunization. This situation is alarming as the routine immunization coverage in Somalia was already low [2]. The country also has one of the smallest number of health workers in the world—fewer than 1 per 1000 population [3]. The number of health care workers in Somalia who contracted COVID-19 remains unknown. However, earlier estimates suggest that during the first 180 days of the outbreak, health workers had a 61% positivity rate and accounted for at least 5% of cases during the epidemic’s peak in the country [3]. This is comparable to the finding of an earlier scoping review suggesting that 3.9% of COVID-19 cases worldwide might have been health workers during the early days of the COVID-19 pandemic [4].

Despite the fragile health system in Somalia because of protracted conflicts, the country was one of the few in Africa that was able to start delivering COVID-19 vaccines (AstraZeneca) on 17 March 2021 [5]. In line with the recommendations of the Strategic Advisory Group of Experts on Immunization (SAGE), Somalia prioritized the following groups to receive the COVID-19 vaccines in the first phase of delivery: health workers; elderly people (older than 65 years); people with pre-existing conditions; and other frontline workers. As of 31 December 2022, 8.5 million doses of COVID-19 vaccines had been administered, and 41.7% of the population had been fully vaccinated [6]. Because of the lack of census data on the health workforce in the country, uptake of the COVID-19 vaccine in health workers is not known. However, a study in 2022 suggested that the overall coverage of the COVID-19 vaccine in health workers in Somalia was 37.4% [7], lower than the other priority groups. The reasons for this low acceptance are not known. Concerns about vaccine safety, efficacy, and potential side effects have been reported to be the main reasons for COVID-19 vaccination hesitancy in health workers [8].

As health workers are generally seen as trustworthy, if they refuse immunization, vaccine hesitancy can spread to the general public. Therefore, understanding the reasons for vaccine acceptance and hesitancy in health workers is important so as not to undermine public confidence [9]. Furthermore, it is important to protect and safeguard these frontline workers in order to avoid disruption of essential health services.

The low vaccine acceptance of the general population and health workers in fragile countries such as Somalia and in sub-Saharan Africa in general is a grave concern [10], because vaccine hesitancy has been identified as a substantial obstacle to effective and long-term control of vaccine-preventable diseases in any country setting [11].

The roll-out of COVID-19 vaccines in Somalia was the first time that mass immunization for the adult population had been undertaken in the country. Given this lack of experience of mass immunization of adults, the country struggled to achieve optimal vaccination coverage in both health workers and the general population. Against this background, the current study aimed to identify factors associated with COVID-19 vaccine acceptance as well as predictors of vaccine hesitancy among health workers in Somalia one year after the introduction of COVID-19 vaccines in the country. For the purpose of this study, vaccine hesitancy was defined as the “delay in acceptance or refusal of vaccination despite the availability of vaccination services” [12]. At the time of this study, the vaccines used in Somalia were AstraZeneca, Johnson & Johnson, and Sinopharm.

## 2. Methods

### 2.1. Study Design and Participants

This was a national cross-sectional study conducted among health workers in Somalia during the initial phase of national deployment of COVID-19 vaccines. Preparation for the study started in June 2021 (collecting names of health care workers, developing and pilot-testing and refining the questionnaire). Data were collected from February to March 2022 among all categories of health worker (doctors, nurses, midwives, pharmacists, specialists, technologists (laboratory and radiology), and primary health care staff) in both the public and private health sectors.

Health workers in six states—Hirshabelle, Jubaland, Puntland, South West, Galmudug, and Banadir Regional Administration, collectively known as the Federal Member States—participated in this study. Because of the lack of national data on the health workforce in the country, the lists of health workers were obtained from the state health administrations.

### 2.2. Sample Size

The minimum sample size was determined based on the formula: *n* = Z^2^∗*P*∗(1 − *P*)/d^2^), where *n* = minimum sample size, Z = confidence level corresponding to 5% significance level (1.96), *P* = our estimated proportion of health workers who were vaccine hesitant (20%), and d = margin of error, set at 0.05. Therefore, the minimum sample size (*n*) was 246 for each of the six states, giving an overall sample of 1476 health workers.

The names of 2352 health workers were obtained from the lists provided by the state health authorities. This number does not necessarily represent the actual number of health workers in the country. Many private health care facilities in remote places are missing from this listing. All of these health workers were contacted and invited to participate in the survey, 311 of whom declined to participate, giving a response rate 86.8%. From the 2041 health workers who were willing to be interviewed, 246 from each state were randomly selected using a random numbers table, to reach the sample size of 1476.

**Figure 1:**
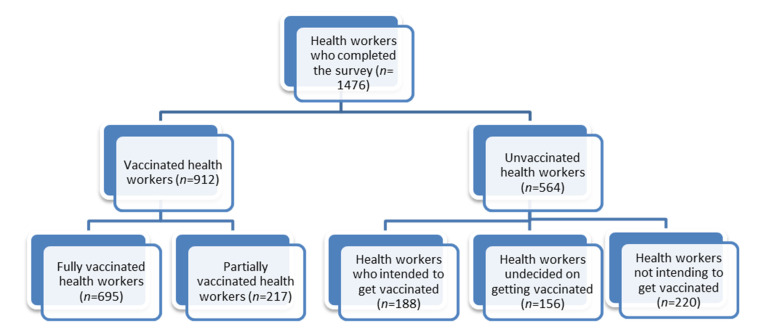
COVID-19 vaccination status of health workers included in the study.

Health workers were included irrespective of their vaccination status. Figure 1 shows the vaccination status of the 1476 health workers.

### 2.3. Data Collection

Development and pilot testing of a structured and closed-ended questionnaire started in June 2021, and ethical clearance for the study was obtained. The questionnaire was adapted from the behavioral and social drivers of vaccination model of the World Health Organization (WHO) and was available in English and Somali [13,14,15]. It was tested on a group of health workers to determine its clarity and applicability. Issues identified during this process were addressed in the final version of the questionnaire used for data collection.

Data were collected from 20 February to 21 March 2022 by 30 trained data collectors using the questionnaire. The data collectors visited all six states and interviewed each of the health workers face-to-face after receiving their informed consent to participate in the study. The interviews were conducted in Somali. No interview was done over the telephone. The questionnaire had five sections: (i) participants’ sociodemographic characteristics (such as, age, sex, profession, marital status, education, and work setting); (ii) history of COVID-19 and other health-related information such as underlying chronic health conditions; (iii) perceived concerns about the COVID-19 vaccine, including views on its safety and effectiveness; (iv) sources of information on COVID-19 vaccines that the health worker thought credible and trusted; and (v) intention to get a COVID-19 vaccine for unvaccinated health workers. For part (v), the health workers were asked, “If not vaccinated, do you intend to get vaccinated?” and the responses were “Yes”, “No”, and “I am currently undecided”. Date were collected using the KoboToolbox mobile data collection platform.

We also included a question on perceived negative treatment because of being a health worker. In some sub-Saharan countries it has been reported anecdotally that health workers were stigmatized and seen as the source of COVID-19 virus transmission because of the nature of their work and their exposure to COVID-19 patients. We aimed to understand if health workers in our study thought that they were stigmatized. If this were the case, this might influence health workers’ decision to accept the COVID-19 vaccine.

For the purpose of this analysis, health workers were considered to be vaccinated if they had received two doses of a COVID-19 vaccine or had received a single dose and were waiting for the second dose. A third (booster) dose was not mandatory in Somalia at the time as many high-risk populations were yet to complete their primary vaccines. Respondents were defined as COVID-19 vaccine hesitant if they had refused or delayed accepting the COVID-19 vaccine despite its availability at designated centers or health facilities where they worked; hence, they were unvaccinated at the time of the survey [12].

### 2.4. Data Analysis

Data review and cleaning were done with Microsoft Excel, input errors were removed, and statistical analysis was conducted using SPSS, version 27 (IBM Corp., Armonk, NY, USA).

Sociodemographic data were summarized as categorical variables, disaggregated by vaccination status. Descriptive statistics (mean and standard deviation (SD), frequency, and percentage) are presented. Sociodemographic characteristics of the health workers who were vaccinated were compared with the characteristics of those who were not vaccinated using the chi-squared test.

We conducted a bivariate analysis with hesitancy as the dependent variable and sociodemographic characteristics as the independent variables. We combined health workers who were undecided and who did not plan to get vaccinated as a single category called “vaccine hesitant” [12]. We compared the characteristics of health workers in the vaccine-hesitant group with those who said that they were willing to get vaccinated at a later time. The results were presented as odds ratios (OR) and 95% confidence intervals (CI). A *p*-value of <0.05 was considered statistically significant.

A multivariable logistic regression analysis was also conducted to assess predictors of vaccine hesitancy in the health workers. The health workers who were not vaccinated at the time of this study, irrespective of their intention to do so at a later stage, were classified as vaccine hesitant (which is consistent with the WHO definition of vaccine hesitancy). We included all clinically important variables in our model. Results are presented as adjusted OR (aOR) with 95% CIs. A *p*-value of <0.05 was considered statistically significant.

### 2.5. Ethical Considerations

The study was approved by the Federal Ministry of Health & Human Services of the Government of Somalia. Data collection was anonymous, and participants’ identities were kept confidential. The participants were informed of the study’s objectives and assured of the anonymity and confidentiality of their data. Written informed consent was collected from the participants before starting the questionnaire.

## 3. Results

### 3.1. Sociodemographic Characteristics

Table 1 shows the sociodemographic characteristics of the 1476 health workers according to their vaccination status. The mean (SD) age of the participants was 34 (11.8) years. The median age was 30 years (interquartile range (IQR) 25–38). A significant difference was seen between the median age of those who were vaccinated and those who were not vaccinated (32 (IQR: 26–39) years versus 28 (IQR: 25–36 years); *p* < 0.001). Most participants were married (61.0%; 900/1476), and 50.3% (743/1476) were men. Nurses made up the greatest proportion of the participants (28.7%; 423/1476), followed by primary health care workers (28.2%; 416/1476). Most participants (62.1%; 916/1476) had a bachelor’s degree as their highest qualification. Just over half of the participants (50.8%; 750/1476) worked in private health facilities, with 49.2% (726/1476) working in the public health sector.

### 3.2. Vaccination Status by Sociodemographic Characteristics

At the time of the study, 61.8% (912/1476) of the health workers were vaccinated (had either had two doses of a COVID-19 vaccine or had a single dose and were waiting for the second dose), while 38.2% (564/1476) were unvaccinated. Significant differences were found between the vaccinated and unvaccinated health workers for all the variables examined except for sex (Table 1).

Among the unvaccinated health workers, 30.3% (171/564) were nurses, followed by primary health care workers (25.5%; 144/564). Among the vaccinated health workers, the greatest proportion was primary health care workers (29.8%; 272/912). Of the vaccinated health workers, 71.4% (651/912) had previously treated a patient with COVID-19, while among unvaccinated health workers, only 42.0% (236/564) had treated a patient with COVID-19. In addition, 78.6% (717/912) of vaccinated health workers had received training on COVID-19 compared with 57.4% (324/564) of unvaccinated health workers. A significantly greater proportion of vaccinated health workers said had been treated negatively because they were a health worker than unvaccinated health workers: 62.4% (569/912) versus 35.6% (201/564).

Among health workers, midwives were the most vaccine hesitant, as 46.8% (89/190) were unvaccinated, while physicians were the least hesitant, with 30.6% (56/183) unvaccinated.

### 3.3. Exposure to COVID-19

As shown in Table 2, 54.7% (807/1476) of health workers had had COVID-19. Of these respondents, 82.8% (668/807) had been confirmed positive for severe acute respiratory syndrome coronavirus 2 (SARS-CoV-2) in a laboratory test. The remaining 17.2% were not specifically asked why they thought they had had COVID-19 as this was not a focus of our study.

Most health workers (60.1%; 887/1476) said that they had cared for or treated a patient with COVID-19, and 60.8% (898/1476) said that they had been in contact with a friend or family member who was positive for COVID-19.

### 3.4. Perceptions of Health Workers about COVID-19 Vaccines

Among vaccinated health workers, 60.7% (554/912) had concerns about the vaccine, compared with 54.6% (308/564) of unvaccinated health workers (*p* = 0.02; Table 3). Only 20.7% (117/564) of unvaccinated health workers thought that the COVID-19 vaccine was very important for their health, compared with 44.5% (406/912) of the vaccinated group (*p* < 0.001).

The perception of vaccinated and unvaccinated health workers about the protection to others if they are vaccinated differed significantly. Most vaccinated health workers (53.9%; 492/912) thought that being vaccinated would also help protect others, compared with 36.9% (208/564) of the unvaccinated group (*p* < 0.001). A significantly greater proportion of vaccinated health workers (41.0%; 374/912) thought the COVID-19 vaccine was very safe than the unvaccinated group (15.6%; 88/564; *p* < 0.001). In addition, 82.0% (748/912) of vaccinated health workers said that they would recommend the COVID-19 vaccine to eligible patients, compared with 54.1% (305/564) in the unvaccinated group (*p*< 0.001).

### 3.5. Factors Associated with COVID-19 Vaccine Hesitancy in Unvaccinated Health Workers

Among the vaccine-hesitant (unvaccinated) health workers, 61.0% (344/564) were willing to accept the vaccine, and 39.0% (220/564) were not willing or were undecided (Table 4). Vaccine hesitancy was significantly associated with: residence in Hirshabelle state (OR = 3.51, 95% CI: 2.07–5.96); working in private health facilities (OR = 1.67, 95% CI: 1.16–2.40); being a primary health care worker (OR = 2.00, 95% CI: 1.07–3.76); having no previous history of COVID-19 (OR = 2.59, 95% CI: 1.76–3.79); not having treated or cared for a COVID-19 patient (OR = 2.19, 95% CI: 1.52–3.14); and having received no training on COVID-19 (OR= 1.98, 95% CI: 1.40–2.79). Compared with health workers 60 years and older, those aged 29 years and younger (OR = 0.31, 95% CI: 0.15–0.66) and 30–39 years (OR = 0.42, 95% CI: 0.19–0.91) were significantly less hesitant to get vaccinated with the COVID-19 vaccine. Sex, marital status, and education were not significantly associated with vaccine hesitancy.

### 3.6. Reasons for COVID-19 Vaccine Hesitancy

The two most important reasons cited by the unvaccinated health workers for their unwillingness to take the COVID-19 vaccine were: (i) need for more information about the vaccine’s safety and effectiveness (39.4%; 222/564) and (ii) the perception that the COVID-19 vaccine has side effects (31.7%; 179/564) (Table 5).

### 3.7. Trusted Sources of Information

**Figure 2:**
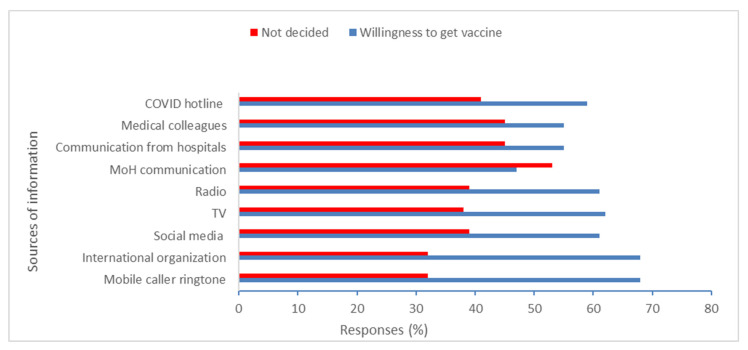
Trusted sources of information on COVID-19 among unvaccinated Somali health workers, by willingness to get vaccinated. Note: Social media included Facebook, Twitter, Snapchat, and TikTok.

Unvaccinated health workers who expressed willingness to get vaccinated reported that mobile telephone caller tune messages (heard by a caller after making an outgoing call to another person), international organizations, and the government COVID-19 hotline were the most trustworthy sources of information about COVID-19 and COVID-19 vaccines that could influence their decision to take the vaccine (Figure 2). Somalia, like many other African and Asian countries, replaced the standard ringtone with COVID-19 awareness and prevention messages when the pandemic started [16]. These messages are in Somali, and the Ministry of Health has approved the content.

### 3.8. Predicators of COVID-19 Vaccine Hesitancy

In the multivariable logistic regression analysis to determine predictors of vaccine hesitancy in health workers, the following factors were significantly associated with hesitancy: being a primary health care worker (adjusted OR (aOR) = 2.37, 95% CI: 1.15–4.90) or nurse (aOR= 2.12, 95% CI: 1.05–4.25) compared with a laboratory technologist; having a master’s degree (aOR = 5.32, 95% CI: 1.28–22.23) compared with a secondary school certificate; living in Hirshabelle State (aOR = 3.23, 95% CI: 1.68–6.20) compared with South West state; not having had COVID-19 (aOR = 1.96, 95% CI: 1.15–3.32); and not having received any training on COVID-19 (aOR = 1.54, 95% CI: 1.02–2.32) (Table 6). No association was found between vaccine hesitancy and perceived negative treatment as a health worker in the regression analysis.

Vaccine hesitancy in health workers was not significantly associated with sex, age, marital status, working in public or private health facilities, having chronic health conditions, and treating or caring for COVID-19 patients (Table 6).

## 4. Discussion

This is the first study in Somalia to report on COVID-19 vaccine acceptance and hesitancy among health care workers in the country and explore the factors associated with their hesitancy. Before the COVID-19 pandemic, Somalia’s immunization program offered only two vaccines for adults—yellow fever for travelers and tetanus toxoid for pregnant women.

Despite health workers being a priority group for vaccination against COVID-19 in the country during the initial phase of vaccine deployment, the acceptance rate, after 12 months of vaccine roll-out, was 61.8% among the health workers. Our study found health workers in Hirshabelle State were significantly more likely to be unvaccinated. This state is one of the newly established states (in 2017), and many parts are inaccessible because they are controlled by insurgent forces. As a result, access to health care is severely disrupted and primary health care services are almost non-existent. Thus, security challenges and other health system problems could be the reason for the poor COVID-19 vaccine coverage in this state.

In the multivariable logistic regression analysis to determine predictors of vaccine hesitancy in health workers, having a master’s degree compared with a secondary school certificate was significantly associated with vaccine hesitancy. It could be that these health workers were delaying vaccination until more data on vaccine safety were available.

Among the vaccine-hesitant health workers, 33.3% were willing to accept the vaccine, and 39.0% were not willing or were undecided. Our study was conducted during the initial phase of vaccine deployment in Somalia, while most published studies available on vaccine hesitancy and acceptance in health care workers around the world predate the deployment of COVID-19 vaccines. For example, in Egypt, a study on COVID-19 vaccination perception and attitudes before the vaccination programme reported that 51% of health workers were undecided about taking the vaccine, 28% were hesitant, and 21% said they would take it [17]. In a similar study in Nigeria before the COVID-19 vaccine was rolled out, the hesitancy rate was 50.5% (95% CI: 45.6–55.3%) [18]. Similarly in Ethiopia, before the COVID-19 vaccine was rolled out, 60.3% of health workers surveyed were vaccine hesitant [19]. However, another study in Nigeria during the initial phase of vaccine deployment found that only 8% health workers interviewed were vaccine hesitant [20]. Our finding of a 61.8% vaccine acceptance rate mirrors the findings of a systematic review of COVID-19 vaccine acceptance in health workers in Africa, which showed that the mean (SD) acceptance rate increased marginally from 55.5% (5.6) in 2021 to 60.8% (5.3) in 2022, resulting in an overall mean COVID-19 vaccine acceptance rate of 58% in Africa in 2022 [21]. The prevalence of vaccine hesitancy in health workers worldwide ranged from 4.3% to 72.0% [11].

This information on COVID-19 vaccine hesitancy and acceptance is important for Somalia. In the absence of any such studies in the past in the general population, our findings will be valuable for understanding and measuring changes over time in the attitude and perception of the general population and health workers regarding COVID-19 vaccines or during the roll-out of other adult vaccines in mass immunization programs. Studies have shown a strong association between vaccination of health workers for COVID-19 and high population coverage. This suggests that achieving high vaccination coverage for COVID-19 and other adult vaccines in health workers by addressing hesitancy could lead to optimal vaccination coverage of the same vaccines in the general population [22].

The two most important reasons cited by the unvaccinated health workers for their hesitancy were the need for more information on the vaccine’s safety and efficacy, and the potential side effects of the COVID-19 vaccine. This is similar to the findings of other studies [21]. Other studies on vaccine hesitancy in health workers found concerns about vaccine safety, efficacy, and side effects were the main reasons for COVID-19 vaccination hesitancy [8].

The vaccinated and unvaccinated health workers in our study differed significantly in their perceptions of the COVID-19 vaccines’ safety and effectiveness. Significantly more vaccinated health workers thought that their getting vaccinated helped protect others and would recommend the COVID-19 vaccine to eligible patients. This is consistent with many studies where the authors have suggested that accessing reliable information, rather than education, was a better determinant of vaccine acceptance [23]. This finding has an important policy implication. Wider dissemination of information using the sources that are most trusted by the health workers in the country would help improve vaccination coverage in health workers and eventually in the general population.

In our study, just over two thirds of health workers reported that mobile telephone ring tone messages were the most trustworthy source of information about COVID-19 and COVID-19 vaccines. Like many other African and Asian countries, Somalia replaced the standard ringtone with COVID-19 awareness and prevention messages. In addition, social media was considered a trusted and credible source of information that could influence health workers’ decision to take the COVID-19 vaccine. This is similar to findings in Ethiopia [19], where health workers frequently viewed social media as the best source of COVID-19 information. Therefore, these sources can be used to amplify messages and develop a risk communication plan that is tailored to address myths and misinformation about COVID-19 vaccine hesitancy and hence contribute to improved coverage.

The factors significantly associated with vaccine hesitancy in our health workers were: being a primary health care worker or a nurse; not having had COVID-19; and not having received any training on COVID-19. This is similar to the findings of other studies [24]. However, unlike another study [25], we did not find any positive association between chronic health conditions and vaccine acceptance. This could be due to the fact that the mean age of our study participants was 34 years, and most reported having no chronic health conditions. Our finding that primary-level health workers and nurses were more likely to be vaccine hesitant needs special attention. Most health workers administering the COVID-19 vaccines as well as managing the essential health services in Somalia belong to these groups. They are also the frontline health workers in the country and are the backbone of COVID-19 vaccine deployment in the country. Therefore, addressing their concerns and hesitancy will serve to advance the vaccination program and improve COVID-19 vaccination coverage in the country, as has been found in Rwanda [26].

Understanding the dynamics of vaccine acceptance and hesitancy has always been important, especially in fragile countries with weak health systems, which are vulnerable to epidemics caused by high-threat pathogens and pandemics such as COVID-19. Vaccines play a key role in ending such epidemics or pandemics. Vaccination of health workers has been shown to protect their families, friends, and patients and reduce infection and transmission in health care settings [27]. However, vaccine hesitancy has been shown to exist among the general population and health workers [28], and it is important to explore interventions to overcome this hesitancy [29]. In our study, being a primary health care worker was associated with vaccine hesitancy. This is a concern as in the national health system of Somalia, primary health care workers are the first contact for a patient. This means that a patient has to go first to a primary health care worker and then to a hospital. Hence, primary health care workers are likely to be in contact with many patients and so are susceptible to infection from COVID-19 patients attending primary health care centers. It is therefore important that they are vaccinated so they do not get sick themselves and do not pass on the infection to primary health care attendees. In addition, they can reach many people to advocate for vaccination. Thus, it is particularly important to tackle hesitancy among primary health care workers. It is therefore vital to ensure that the concerns of Somali health workers about vaccines are addressed and that they are vaccinated.

## 5. Limitations and Strengths

Because of the design of the survey, we could not establish causality nor observe trends over time. In addition, our findings may be susceptible to non-response bias, as health workers who agreed to participate may have disproportionately different characteristics compared with non-respondents. We did not collect data from Somaliland, which represents 25% of the Somali population. Despite these limitations, this study has some strengths. This is the first study in Somalia to determine predictors of vaccine hesitancy in health care workers. Unlike many other studies on vaccine hesitancy that used telephone interviews or web-based surveys, we collected data in face-to-face interviews conducted by trained interviewers. Hence, there were opportunities to cross-check responses during the interview, limiting the biases. In addition, this study was conducted at a time when the COVID-19 vaccines were being rolled out in the country, so the findings on acceptance and hesitancy are from real-life experience.

## 6. Conclusions

The findings of this study present a unique opportunity to the policy planners and public health officials in the country to address the factors contributing to or associated with vaccine hesitancy and improve COVID-19 vaccine uptake among both health workers and the general public. The findings can be used to help improve vaccination coverage in health workers for any adult vaccine in the future, such as seasonal influenza and other pandemic vaccines, and hence help enhance Somalia’s epidemic and pandemic readiness.

## Figures and Tables

**Table 1:** Sociodemographic characteristics of study participants.

Sociodemographic Characteristic	Total, N (%)	Vaccinated, N (%)	Not Vaccinated, N (%)	*p* *
	*n =* 1476	*n* = 912 (61.8)	*n =* 564 (38.2)	
Sex				
Female	733 (49.7)	439 (48.1)	294 (52.1)	0.136
Male	743 (50.3)	473 (51.9)	270 (47.9)	
Age, in years				
Mean (SD)	34 (11.8)	35 (11.8)	32 (11.6)	<0.001
Median (IQR)	30 (25–38)	32 (26–39)	28 (25–36)	
<30	696 (47.2)	379 (41.6)	317 (56.2)	
30–39	455 (30.8)	306 (33.6)	149 (26.4)	
40–49	158 (10.7)	111 (12.2)	47 (8.3)	
50–59	79 (5.4)	60 (6.6)	19 (3.4)	
>59	88 (6)	56 (6.1)	32 (5.7)	
Marital status				
Married	900 (61.0)	589 (64.6)	311 (55.1)	<0.001
Single	576 (39.0)	323 (35.4)	253 (44.9)	
Education				
Doctorate	1 (0.1)	1 (0.1)	0 (0.0)	<0.001
Master’s degree	79 (5.4)	65 (7.1)	14 (2.5)	
Bachelor’s degree	916 (62.1)	609 (66.8)	307 (54.4)	
Diploma	313 (21.2)	157 (17.2)	156 (27.7)	
Secondary school certificate	167 (11.3)	80 (8.8)	87 (15.4)	
Occupation				
Primary health care worker	416 (28.2)	272 (29.8)	144 (25.5)	0.002
Other health care worker **	123 (8.3)	83 (9.1)	40 (7.1)	
Technician (laboratory and radiology)	141 (9.6)	77 (8.4)	64 (11.3)	
Midwife	190 (12.9)	101 (11.1)	89 (15.8)	
Nurse	423 (28.7)	252 (27.6)	171 (30.3)	
Physician	183 (12.4)	127 (13.9)	56 (9.9)	
State				
Banadir Regional Administration	246 (16.6)	216 (23.7)	30 (5.3)	0.001
Galmudug State	246 (16.6)	146 (16.0)	100 (17.7)	
Hirshabelle State	246 (16.6)	89 (9.8)	157 (27.8)	
Jubaland State	246 (16.6)	132 (14.5)	114 (20.2)	
Puntland	246 (16.6)	187 (20.5)	59 (10.5)	
South West State	246 (16.6)	142 (15.6)	104 (18.4)	
Institution				
Private	750 (50.8)	384 (42.1)	366 (64.9)	0.001
Public	726 (49.2)	528 (57.9)	198 (35.1)	
Chronic health conditions				
No	866 (58.7)	483 (53.0)	383 (67.9)	<0.001
Yes	565 (38.3)	404 (44.3)	161 (28.5)	
Not sure	45 (3.0)	25 (2.7)	20 (3.5)	
Treated a patient with COVID-19				
No	522 (35.4)	228 (25.0)	294 (52.1)	
Yes	887 (60.1)	651 (71.4)	236 (42.0)	0.001
Don’t know	67 (4.5)	33 (3.6)	34 (6.0)	
Treated negatively as a health worker				
No	590 (40.0)	299 (32.8)	291 (51.6)	0.001
Yes	770 (52.2)	569 (62.4)	201 (35.6)	
Not sure	116 (7.9)	44 (4.8)	72 (12.8)	
Received training on COVID-19				
No	435 (29.5)	195 (21.4)	240 (42.6)	0.001
Yes	1041 (70.5)	717 (78.6)	324 (57.4)	

SD: standard deviation; IQR: interquartile range. * Chi-squared test, significant at *p* < 0.05. ** Includes anaesthesiologists; dentists; ear, nose, and throat specialists; gynaecologists; nutritionists; hospital directors; nutritionists; ophthalmologists; and pharmacists.

**Table 2:** Exposure to COVID-19.

Question	N (%)
To your knowledge, have you ever had COVID-19? (*n =* 1476)	
Yes	807 (54.7)
No	572 (38.8)
Not sure	97 (6.6)
If yes, was COVID-19 confirmed by a test? (*n =* 807)	
Yes	668 (82.8)
No	139 (17.2)
Have you been in contact with or treated a COVID-19 patient? (*n =* 1476)	
Yes	887 (60.1)
No	522 (35.4)
Don’t know	67 (4.5)
Did anyone of your family, friends or co-workers have COVID-19? (*n =* 1476)	
Yes	898 (60.8)
No	362 (24.5)
Not sure	201 (13.6)
Prefer not to say	15 (1.0)

**Table 3:** Somali health care workers’ perception of the COVID-19 vaccine.

Question	Vaccinated, N (%)	Not vaccinated, N (%)	*p* *
	(*n =* 912)	(*n =* 564)	
Do you have any concerns about the COVID-19 vaccine?			
Yes	554 (60.7)	308 (54.6)	0.02
No	358 (39.3)	256 (45.4)	
How important do you think getting a COVID-19 vaccine is for your health?			
Not at all important	78 (8.6)	68 (12.1)	<0.001
A little important	188 (20.6)	147 (26.1)	
Moderately important	232 (25.4)	123 (21.8)	
Very important	406 (44.5)	117 (20.7)	
Not sure	8 (0.9)	109 (19.3)	
How much do you think getting a COVID-19 vaccine for yourself protects others?			
It will help protect others to some extent	333 (36.5)	161 (28.5)	<0.001
It will help protect others very much	492 (53.9)	208 (36.9)	
It will not make any difference to other people	54 (5.9)	59 (10.5)	
Not sure	33 (3.6)	136 (24.1)	
How safe do you think a COVID-19 vaccine is for you?			
Not at all safe	48 (5.3)	54 (9.6)	<0.001
A little safe	174 (19.1)	150 (26.6)	
Moderately safe	305 (33.4)	157 (27.8)	
Very safe	374 (41.0)	88 (15.6)	
Not sure	11 (1.2)	115 (20.4)	
How concerned are you that a COVID-19 vaccine could cause you to have a serious illness?			
Not at all concerned	173 (19.0)	74 (13.1)	
A little concerned	211 (23.1)	154 (27.3)	<0.001
Moderately concerned	299 (32.8)	132 (23.4)	
Very concerned	207 (22.7)	101 (17.9)	
Not sure	22 (2.4)	103 (18.3)	
Would you recommend a COVID-19 vaccine to eligible patients?			
Yes	748 (82.0)	305 (54.1)	<0.001
No	110 (12.1)	154 (27.3)	
Not sure	54 (5.9)	105 (18.6)	
Do you think most health workers you know will get the COVID-19 vaccine?			
Yes	704 (77.2)	246 (43.6)	<0.001
No	112 (12.3)	160 (28.4)	
Not sure	96 (10.5)	158 (28.0)	
Have you heard anything negative about COVID-19 vaccines?			
Yes	635 (69.6)	322 (57.1)	<0.001
No	230 (25.2)	168 (29.8)	
Not sure	47 (5.2)	74 (13.1)	

* Chi-squared test significant at *p* < 0.05.

**Table 4:** Bivariate analysis of the hesitancy and willingness of unvaccinated Somali health workers to take the COVID-19 vaccine.

Variable	Hesitant (*n* = 220) N (%)	Willing (*n* = 344) N (%)	p *	OR (95% CI)
Sex				
Female	106 (48.2)	188 (54.7)	0.13	0.77 (0.55–1.08)
Male	114 (51.8)	156 (45.3)		1
Age, in years				
<30	108 (49.1)	209 (60.7)	0.004	0.31 (0.15–0.66)
30–39	61 (27.7)	88 (25.6)		0.42 (0.19–0.91)
40–49	19 (8.6)	28 (8.1)		0.41 (0.16–1.03)
50–59	12 (5.5)	7 (2)		1.03 (0.32–3.33)
≥60	20 (9.1)	12 (3.5)		1
State				
Banadir Regional Administration	7 (23.3)	23 (76.7)	0.001	0.72 (0.28–1.84)
Galmudug State	31 (31)	69 (69)		1.06 (0.58–1.92)
Hirshabelle State	94 (42.7)	63 (18.3)		3.51 (2.07–5.96)
Jubaland State	45 (20.5)	69 (20.1)		1.54 (0.87–2.70)
Puntland	12 (5.5)	47 (13.7)		0.60 (0.28–1.29)
South West State	31 (14.1)	73 (21.2)		1
Institution				
Private	158 (71.8)	208 (60.5)	0.001	1.67 (1.16–2.40)
Public	62 (28.2)	136 (39.5)		1
Marital status				
Married	126 (57.3)	185 (53.8)	0.416	1.15 (0.81–1.62)
Single	94 (42.7)	159 (46.2)		1
Education				
Master’s degree	9 (4.1)	5 (1.5)	0.108	2.81 (0.87– 9.09)
Bachelor’s degree	110 (50)	197 (57.3)		0.87 (0.53–1.42)
Diploma	67 (30.5)	89 (25.9)		1.17 (0.69– 2)
Secondary school certificate	34 (15.5)	53 (15.4)		1
Occupation				
Primary healthcare worker	66 (30)	78 (22.7)	0.110	2.00 (1.07–3.76)
Other health care worker **	19 (8.6)	21 (6.1)		2.14 (0.94–4.89)
Technician (laboratory and radiology)	19 (8.6)	45 (13.1)		1
Midwife	34 (15.5)	55 (16)		1.46 (0.74–2.91)
Nurse	66 (30)	105 (30.5)		1.49 (0.80–2.76)
Physician	16 (7.3)	40 (11.6)		0.95 (0.43–2.09)
Chronic health condition				
Yes	51 (23.2)	110 (32.0)	0.01	1
No	165 (75.0)	218 (63.4)		1.63 (1.11–2.41)
Not sure	4 (1.8)	16 (4.7)		0.54 (0.172–1.69
Had COVID-19				
Yes	54 (24.5)	149 (43.3)	<0.001	1
No	148 (67.3)	158 (45.9)		2.59 (1.76–3.79)
Not sure	18 (18.2)	37 (10.8)		1.34 (0.71–2.56)
Treated or cared for COVID-19 patient				
Yes	70 (31.8)	166 (48.3)	<0.001	1
No	141 (64.1)	153 (44.5)		2.19 (1.52–3.14)
Don’t know	9 (4.1)	25 (7.3)		0.85 (0.38–1.92)
Received training on COVID-19				
Yes	104 (47.3)	220 (64)	<0.001	1
No	116 (52.7)	124 (36)		1.98 (1.40–2.79)

OR: odds ratio; CI: confidence interval. * Chi-squared test significant at *p* < 0.05. ** Includes anaesthesiologists; dentists; ear, nose, and throat specialists; gynaecologists; nutritionists; hospital directors; nutritionists; ophthalmologists; and pharmacists.

**Table 5:** Reasons for COVID-19 vaccine hesitancy in the unvaccinated Somali health workers.

Reason	N (%) (*n* = 564)
Need further information about the COVID-19 vaccine	222 (39.4)
The COVID-19 vaccine has side effects	179 (31.7)
Require family permission before taking the COVID-19 vaccine	35 (6.2)
Pregnancy	30 (5.3)
Belief that there is a shortage of COVID-19 vaccines	19 (3.4)
Prefer to wait for another type of COVID-19 vaccine	15 (2.7)
Concern over vaccine efficacy	15 (2.7)
Not a priority	12 (2.1)
No time because of workload	11 (2.0)
Other reasons	8 (1.4)
The vaccine not permitted religiously	6 (1.1)
Circulating rumors in the community	6 (1.1)
Not eligible yet to get the COVID-19 vaccine	3 (0.5)
The vaccine is painful to get	3 (0.5)

Note: The total percentage is not 100% because multiple responses were given.

**Table 6:** Multivariable logistic regression analysis of predictors of COVID-19 vaccine hesitancy among Somali health workers.

Variables	*p*	Adjusted OR (95% CI) *
Sex		
Female	0.476	0.85 (0.55–1.33)
Male		1
Age, in years		
<30	0.105	0.48 (0.20–1.17)
30–39	0.292	0.61 (0.24–1.53)
40–49	0.079	0.39 (0.13–1.12)
50–59	0.666	1.33 (0.36–4.93)
≥60		1
Marital status		
Married	0.236	1.33 (0.83–2.13)
Single *		1
Education		
Secondary school certificate		1
Bachelor’s degree	0.484	1.25 (0.67–2.34)
Diploma	0.314	1.37 (0.74–2.52)
Master’s degree	0.022	5.32 (1.28–22.23)
Occupation		
Primary health care worker	0.020	2.37(1.15–4.90)
Other health care worker	0.052	2.50 (0.99–6.34)
Technician (laboratory and radiology)		1
Midwife	0.064	2.19 (0.96–4.02)
Nurse	0.035	2.12 (1.05–4.25)
Physician	0.447	1.43 (0.57–3.60)
State		
South West		1
Banadir	0.599	0.76 (0.27–2.15)
Galmudug	0.516	1.26 (0.63–2.54)
Hirshabelle	<0.001	3.23 (1.68–6.20)
Jubaland	0.715	1.13 (0.58–2.19)
Puntland	0.201	0.55 (0.21–1.38)
Institution		
Private	0.443	0.83 (0.52–1.34)
Public		1
Chronic health conditions		
Yes		1
No	0.677	1.12 (0.67–1.87)
Not sure	0.473	0.63 (0.18–2.23)
Treated or cared for COVID-19 patient		
Yes		1
No	0.128	1.44 (0.90–2.30)
Don’t know	0.750	0.85 (0.318–2.29)
Had COVID-19		
Yes		1
No	0.013	1.96 (1.15–3.32)
Not sure	0.326	1.52 (0.66–3.47)
Received training on COVID-19		
Yes		1
No	0.041	1.54 (1.02–2.32)

OR: odds ratio; CI: confidence interval. * Adjusted for all the other variables in the table.

## Data Availability

The data presented in this study are available in the article.

## References

[1] World Health Organization COVID-19 Information Note 13. Disruption of Essential Health Services and Its Impact in Somalia: What We Know So Far. https://www.emro.who.int/images/stories/somalia/documents/covid-19-information-note-13.pdf.

[2] World Health Organization COVID-19 Information Note 20. Accelerated Immunization Campaign for COVID-19 and Childhood Vaccines, Somalia, November 2021–January 2022: Progress and Lessons Learnt. https://www.emro.who.int/images/stories/somalia/documents/covid-19-information-note-20.pdf.

[3] Abdi A., Ahmed A.Y., Abdulmunim M., Karanja M.J., Solomon A., Muhammad F., Kumlachew M., Obtel M., Malik S.M.M.R. (2021). Preliminary findings of COVID-19 infection in health workers in Somalia: A reason for concern. Int. J. Infect. Dis..

[4] Bandyopadhyay S., Baticulon R.E., Kadhum M., Alser M., Ojuka D.K., Badereddin Y., Kamath A., Parepalli S.A., Brown G., Iharchane S. (2020). Infection and mortality of healthcare workers worldwide from COVID-19: A systematic review. BMJ Glob. Health.

[5] Hamayoun M., Abdulrazak I., Farid M., Malik M.R., Mohamud M.F. (2022). Adverse events following introduction of the ChAdOx1 nCoV-19 vaccine in Somalia in 2021: Findings from a fragile setting and implications for the future. IJID Reg..

[6] World Health Organization COVID-19 Information Note 23. Accelerated COVID-19 Vaccination Campaign, Somalia September–December 2022: 41.7% People Fully Vaccinated. https://www.emro.who.int/images/stories/somalia/documents/covid-19-information-note-23.pdf?ua=1.

[7] Dahie H.A., Mohamoud J.H., Adam M.H., Garba B., Dirie N.I., Sh Nur M.A., Mohamed F.Y. (2022). COVID-19 vaccine coverage and potential drivers of vaccine uptake among healthcare workers in Somalia: A cross-sectional study. Vaccines.

[8] Biswas N., Mustapha T., Khubchandani J., Price J.H. (2021). The nature and extent of COVID-19 vaccination hesitancy in healthcare workers. J. Community Health.

[9] Huang D., Ganti L., Graham E.W., Shah D., Aleksandrovskiy I., Al-Bassam M., Fraunfelter F., Falgiani M., Leon L., Lopez-Ortiz C. (2022). COVID-19 vaccine hesitancy among healthcare providers. Health Psychol. Res..

[10] Kabakama S., Konje E.T., Dinga J.N., Kishamawe C., Morhason-Bello I., Hayombe P., Adeyemi O., Chimuka E., Lumu I., Amuasi J. (2022). Commentary on COVID-19 vaccine hesitancy in sub-Saharan Africa. Trop. Med. Infect. Dis..

[11] Omer S.B., Salmon D.A., Orenstein W.A., Dehart M.P., Halsey N. (2009). Vaccine refusal, mandatory immunization, and the risks of vaccine-preventable diseases. N. Engl. J. Med..

[12] MacDonald N.E. (2015). Vaccine hesitancy: Definition, scope and determinants. Vaccine.

[13] Alagarsamy S., Mehrolia S., Pushparaj U., Jeevananda S. (2022). Explaining the intention to uptake COVID-19 vaccination using the behavioral and social drivers of vaccination (BeSD) model. Vaccine X.

[14] WHO (2022). Understanding the behavioural and social drivers of vaccine uptake: WHO position paper—May 2022. Wkly. Epidemiol. Rec..

[15] Jiang B., Cao Y., Qian J., Jiang M., Huang Q., Sun Y., Dai P., Yi H., Zhang R., Xu L. (2023). Healthcare workers attitudes toward influenza vaccination: A behaviour and social drivers survey. Vaccines.

[16] Appiah B., Asamoah-Akuoko L., France C., Rene A., Amanquah N., Bates I. (2022). Pharmacists and COVID-19 vaccination—Considering mobile phone caller tunes as a novel approach to promote vaccine uptake in low-and middle-income countries. Res. Soc. Adm. Pharm..

[17] Fares S., Elmnyer M.M., Mohamed S.S., Elsayed R. (2021). COVID-19 vaccination perception and attitude among healthcare workers in Egypt. J. Prim. Care Community Health.

[18] Amuzie C.I., Odini F., Kalu K.U., Izuka M., Nwamoh U., Emma-Ukaegbu U., Onyike G. (2021). COVID-19 vaccine hesitancy among healthcare workers and its socio-demographic determinants in Abia State, Southeastern Nigeria: A cross-sectional study. Pan Afr. Med. J..

[19] Mohammed R., Nguse T.M., Habte B.M., Fentie A.M., Gebretekle G.B. (2021). COVID-19 vaccine hesitancy among Ethiopian healthcare workers. PLoS ONE.

[20] Nomhwange T., Wariri O., Nkereuwem E., Olanrewaju S., Nwosu N., Adamu U., Danjuma E., Onuaguluchi N., Enegela J., Nomhwange E. (2022). COVID-19 vaccine hesitancy amongst healthcare workers: An assessment of its magnitude and determinants during the initial phase of national vaccine deployment in Nigeria. eClinicalMedicine.

[21] Njoga E.O., Awoyomi O.J., Onwumere-Idolor O.S., Awoyomi P.O., Ugochukwu I.C., Ozioko S.N. (2022). Persisting vaccine hesitancy in Africa: The whys, global public health consequences and ways-out—COVID-19 vaccination acceptance rates as case-in-point. Vaccines.

[22] Fotiadis K., Dadouli K., Avakian I., Bogogiannidou Z., Mouchtouri V.A., Gogosis K., Speletas M., Koureas M., Lagoudaki E., Kokkini S. (2021). Factors associated with healthcare workers (HCWs) acceptance of COVID-19 vaccinations and indications of a role model towards population vaccinations from a cross-sectional survey in Greece, May 2021. Int. J. Environ. Res. Public Health.

[23] Ciardi F., Menon V., Jensen J.L., Shariff M.A., Pillai A., Venugopal U., Kasubhai M., Dimitrov V., Kanna B., Poole B.D. (2021). Knowledge, attitudes and perceptions of COVID-19 vaccination among healthcare workers of an inner-city hospital in New York. Vaccines.

[24] Dror A.A., Eisenbach N., Taiber S., Morozov N.G., Mizrachi M., Zigron A., Srouji S., Sela E. (2020). Vaccine hesitancy: The next challenge in the fight against COVID-19. Eur. J. Epidemiol..

[25] Janssen C., Maillard A., Bodelet C., Claudel A.-L., Gaillat J., Delory T., Group A.A.S. (2021). Hesitancy towards COVID-19 vaccination among healthcare workers: A multi-centric survey in France. Vaccines.

[26] Robson J., Bao J., Wang A., McAlister H., Uwizihiwe J.-P., Sayinzoga F., Sibomana H., Koswin K., Wong J., Zlotkin S. (2020). Making sense of Rwanda’s remarkable vaccine coverage success. Int. J. Healthc..

[27] Waldman S.E., Buehring T., Escobar D.J., Gohil S.K., Gonzales R., Huang S.S., Olenslager K., Prabaker K.K., Sandoval T., Yim J. (2022). Secondary cases of Delta variant coronavirus disease 2019 among vaccinated healthcare workers with breakthrough infections is rare. Clin. Infect. Dis..

[28] Yasmin F., Najeeb H., Moeed A., Naeem U., Asghar M.S., Chughtai N.U., Yousaf Z., Seboka B.T., Ullah I., Lin C.Y. (2021). COVID-19 Vaccine hesitancy in the United States: A systematic review. Front. Public Health.

[29] Ryan J., Malinga T. (2021). Interventions for vaccine hesitancy. Curr. Opin. Immunol..

